# Utility of a machine-guided tool for assessing risk behaviour associated with contracting HIV in three sites in South Africa

**DOI:** 10.1016/j.imu.2023.101192

**Published:** 2023

**Authors:** M. Majam, B. Segal, J. Fieggen, Eli Smith, L. Hermans, L. Singh, M. Phatsoane, L. Arora, S.T. Lalla-Edward

**Affiliations:** a Ezintsha, Faculty of Health Sciences, University of the Witwatersrand, Johannesburg, South Africa; b Phithos Technologies, Johannesburg, South Africa; c Department of Microbiology, University Medical Center Utrecht, Utrecht, the Netherlands; d Infectious Diseases Unit, Department of Medicine, University of Cape Town, Cape Town, South Africa

**Keywords:** Machine guided tool, Machine learning, ML, Supervised learning, HIV, HIV risk Assessment, PrEP, Predictive analysis

## Abstract

**Introduction:**

Digital data collection and the associated mobile health technologies have allowed for the recent exploration of artificial intelligence as a tool for combatting the HIV epidemic. Machine learning has been found to be useful both in HIV risk prediction and as a decision support tool for guiding pre-exposure prophylaxis (PrEP) treatment. This paper reports data from two sequential studies evaluating the viability of using machine learning to predict the susceptibility of adults to HIV infection using responses from a digital survey deployed in a high burden, low-resource setting.

**Methods:**

1036 and 593 participants were recruited across two trials. The first trial was a cross-sectional study in one location and the second trial was a cohort study across three trial sites. The data from the studies were merged, partitioned using standard techniques, and then used to train and evaluate multiple different machine learning models and select and evaluate a final model. Variable importance estimates were calculated using the PIMP and SHAP methodologies.

**Results:**

Characteristics associated with HIV were consistent across both studies. Overall, HIV positive patients had a higher median age (34 [IQR: 29–39] vs 26 [IQR 22–33], p < 0.001), and were more likely to be female (155/703 [22%] vs 107/927 [12%], p < 0.001). HIV positive participants also had more commonly gone a year or more since their last HIV test (183/262 [70%] vs 540/1368 [39%], p < 0.001) and were less likely to report consistent condom usage (113/262 [43%] vs 758/1368 [55%], p < 0.001). Patients who reported TB symptoms were more likely to be HIV positive. The trained models had accuracy values (AUROCs) ranging from 78.5% to 82.8%. A boosted tree model performed best with a sensitivity of 84% (95% CI 72–92), specificity of 71% (95% CI 67–76), and a negative predictive value of 95% (95% CI 93–96) in a hold-out dataset. Age, duration since last HIV test, and number of male sexual partners were consistently three of the four most important variables across both variable importance estimates.

**Conclusions:**

This study has highlighted the synergies present between mobile health and machine learning in HIV. It has been demonstrated that a viable ML model can be built using digital survey data from an low-middle income setting with potential utility in directing health resources.

## 1 Introduction

Ending the HIV epidemic has been the focus of global health efforts for the better part of the last two decades, and the UNAIDS “Fast-track” targets of 95%-95%-95% for HIV testing, treatment, and viral suppression are generally accepted as the foundation for ending the HIV epidemic by 2030 [1,2]. Prioritising geographical locations and populations which are lagging is fundamental to achieving these targets [3]. In addition, identifying groups at high risk of HIV acquisition not only allows for improved testing strategies but also facilitates the implementation of effective preventative strategies such as Pre-Exposure Prophylaxis (PrEP), which has been shown to be up to 100% effective in preventing HIV transmission [[4], [5], [6], [7], [8]].

There has been significant effort to leverage digital technology, including strategies such as rapid diagnostic self-tests, and mobile health (mHealth) and electronic health (eHealth) technologies, to help combat the HIV epidemic [9,10]. More recent strategies have coupled such approaches with advanced data sciences techniques, including machine learning (ML) and artificial intelligence, to augment the digital tools [11]. Such strategies have found particular relevance in quantifying or predicting the risk of acquiring an illness [12,13].

In HIV ML modelling and AI algorithms have been shown to be very effective in classifying and quantifying HIV risk across both high-income and low-middle income (LMIC) settings [[14], [15], [16], [17], [18]]. Such techniques have also found potential utility in decision making around PrEP [19,20], which may be of particular relevance in more resource limited environments [21]. These studies are typically based on secondary data analysis, often using large electronic health record datasets. However, this limits their ability to assess the utility of such techniques when using data collected via mHealth methods that are more appropriate to LMICs.

This paper reports data from two sequential studies undertaken by the authors to evaluate the viability of using ML to predict the susceptibility of adults to HIV infection using responses from a digital survey. Four broad categories of input variables were evaluated: demographics, lifestyle, sexual behaviour, and symptoms, each of which comprised its own set of questions.

## 2 Methods

### 2.1 Objectives

To evaluate the accuracy of a ML-based risk assessment tool, trained using data collected from a digital survey, in assessing HIV risk in those believed to be negative or unaware of their HIV status.

### 2.2 Study design

Two separate studies were conducted. Trial one was a cross sectional study conducted in an urban setting in Johannesburg to evaluate the correlation between self-reported socio-demographic, and behavioural risk factors and HIV status among adults believed to be HIV negative. Some of the initial exploratory data analysis of trial one is reported in the protocol for trial two published in 2021 [22]. Trial two was a longitudinal study in which data were collected in two phases with phase one the first visit and phase two a follow-up three months later.

On recruitment for both trials, participants were educated on the reason for the study and what would be required of them. Thereafter, consenting participants responded to a chain of behavioural questions on a digital application platform independent of assistance from study staff. Exceptions were only made on solicitation by the participant. Some of the questions and/or fields present in the first trial were consolidated or excluded in the second trial to make a more concise and accurate screening tool.

Ground truth was established using two RDT HIV tests (First Response HIV-1-2-0 [Premier Medical Corporation Ltd., Kachigam, India] and Alere Determine HIV1/2 [Alere Medical Co. Ltd, Matsudo, Japan]) performed by a trained nurse/counsellor. Patients who tested HIV positive were advised to access medical intervention at a facility of their choosing. In the event of discordant results, the RDT procedure was repeated. In the second trial, patients who tested HIV negative were invited to present themselves for a second visit three months after the first.

**Fig. 1:**
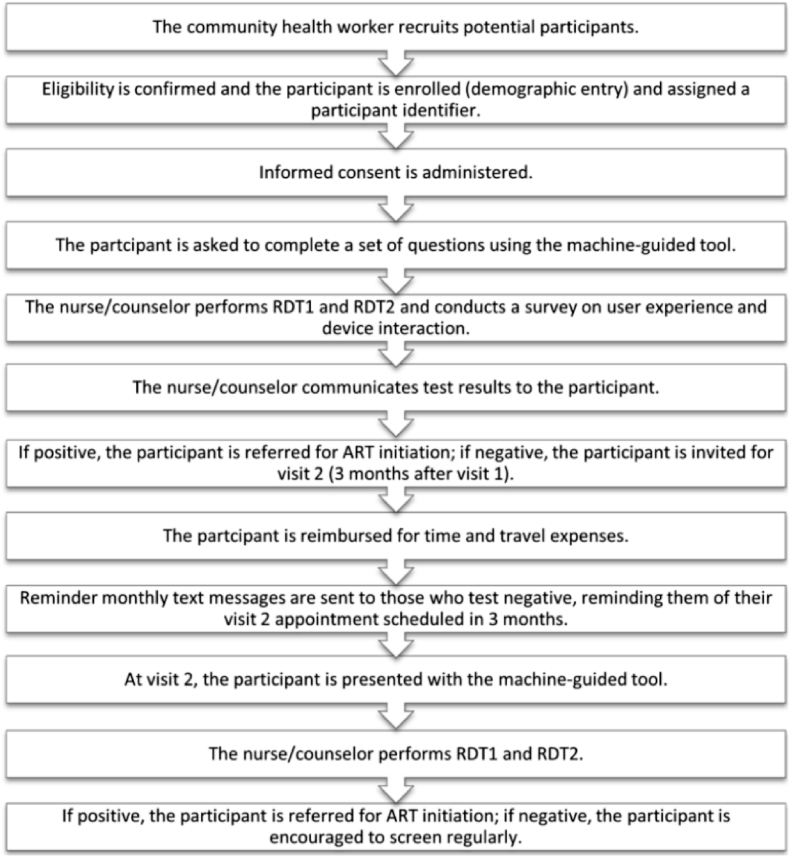
Flowchart of the study process [22]. Abbreviations: RDT, rapid diagnostic test; ART, antiretroviral therapy.

At the end, participants filled out a user experience form after receiving their HIV test results. In the second trial, negative patients were furnished with an appointment card, and then sent monthly text message reminders to present themselves at the second visit. On attending the second visit, the process was repeated. On culmination of this visit, participants who tested positive for HIV were referred for clinical intervention and those who tested negative were advised to maintain regular screening (see Fig. 1).

### 2.3 Study population

Male and female adults who were either self-declared HIV negative or who were of uncertain HIV status from rural, semi-urban and urban populations, were recruited into the study.

### 2.4 Study sites

Study participants were recruited from communities across three provinces. Trial one was conducted in Johannesburg, Gauteng (urban) while trial two was conducted in Tshwane, Gauteng (urban); Gert Sibande, Mpumalanga (semi-urban); and Ugu, Kwa-Zulu Natal (rural).

### 2.5 Sampling and sample size

Non-probability sampling was employed for both trials. Convenience sampling was used for trial one and a combination of convenience and snowball sampling was used for trial two. Phase one was a cross-sectional study and enrolled all consenting patients presenting to the study site. The sample size calculation for phase two was informed by findings from Figueroa et al. [23]. Considering this, a sample size of 600 was estimated with an attempt made to acquire a sample that comprised equal numbers of males and females.

### 2.6 Inclusion and exclusion criteria

#### 2.6.1 Inclusion

The inclusion criteria included adult candidates who could speak and read English as well as demonstrate comprehension of the written informed consent form. In addition, participants had to have a negative or unknown HIV status, be willing to detail their accurate medical history, and supply specimens for two finger-prick blood tests.

#### 2.6.2 Exclusion

The exclusion criteria included known HIV positive status, currently receiving PrEP or antiretroviral treatment, or having received any experimental HIV vaccine. Candidates who, in the estimation of the facilitator, displayed inability to perform the study processes (e.g., acutely ill, under the influence of substances) were also excluded.

### 2.7 Data management

HSTAR staff were responsible for all data management and quality control of both electronic-based and paper-based data. Paper-based data were entered into an electronic database by a data-capturer within two days of having received it from research personnel.

### 2.8 Data analysis

#### 2.8.1 Data cleaning

Data were initially extracted from the relational database used to store responses to the mobile client, and then reformulated to provide human-readable, single row views of each respondent with the associated ground truth test being linked via the unique study identifier. Data were partitioned according to the phase of collection with a single dataset for the first trial and two datasets (initial enrolment and three-month follow-up) for the second trial.

##### 2.8.1.1 Merging of trial data

Collated data were produced by merging the initial presentation datasets from both trials utilising fields common to both. This was possible as the fields collected during the first trial formed a superset of the fields present in the second trial. Fields were merged using human-readable response values. Careful attention was paid to ensuring consistency between the sets so as not to introduce errors secondary to different variable encoding schemas.

#### 2.8.2 Descriptive statistics

Data distributions were assessed using density plots and descriptive statistics presented as per data normality. Hypothesis testing was performed using Chi Squared tests for categorical variables, ANOVA for normal continuous variables, Kruskal-Wallis for non-normal continuous variables, and Fisher's exact tests for categorical variables with small cells (n ≤ 5). Statistical significance was considered below an α value of 0.05 and descriptive statistics were performed in RStudio [24].

#### 2.8.3 Data partitioning and model building

Baseline exploratory data analysis was conducted utilising conventional univariate and multivariable modelling as well as visualisation of the distribution of variables. Subsequent predictive modelling utilised several data partitions. Initial ML model selection employed only data from the first trial with the set randomly partitioned via a 70:30 split into training and test data. Training data were then split repeatedly via 10-fold cross validation for model parameter selection via the R caret library [25]. Area under the receiver operator curve (AUROC) was used as the performance metric for parameter selection with 95% confidence intervals calculated for each via bootstrapping. The final chosen parameters were thereafter fitted on the full training set and finally evaluated on the holdout test set. A variety of models were evaluated including standard logistic Generalised Linear Models (GLMs), Bayesian GLMs, Lasso Regression (LR), Support Vector Machines (SVMs), Decision Trees, and Gradient Boosted Decision Trees [26]. All implementations were derived from the caret package with a fixed data split used across all models. All models utilised only features available in both trial datasets. These overlapping features were encoded in the same manner across all analyses to ensure interoperability between models.

The generalisability of models trained on data from trial one was evaluated using the second trial's data as an external test set. This was then repeated with the second phase of trial two to evaluate the prospective predictive ability of the model in stratifying individuals at risk of seroconversion.

#### 2.8.4 Variable importance

Variable importance was assessed in two ways. The first method used an implementation of the Permutation Importance (PIMP) algorithm [27]. This method employs the final trained model and shuffles individual predictor columns (or groups thereof) and determines the reduction in performance, in this case AUROC, across several replications. This change in performance is compared against a null distribution wherein both a predictor and the outcome columns are shuffled. This produces two distributions with the distance between them indicating the scale of performance difference obtained with inclusion of the given variable. The shuffling operation was repeated a thousand times to produce each distribution. This distribution distance is quantified and compared by means of a T-statistic which is subsequently utilised to produce a set of variables ordered by importance to model predictive performance. In addition, the use of an outcome-linked variable importance methodology was chosen to provide insight into a variable's utility in discriminating between risk groups as opposed to simply attributing predictions to features. To this end, SHapley Additive exPlanations (SHAP) [28] were used to decompose model predictions on aggregate- and individual-level outputs for the purpose of aiding in model explainability a both levels.

### 2.9 Ethical considerations

This study received ethical approval from the University of Witwatersrand HREC (ethics reference no. 200312) in August 2020 and is on the South African National Clinical Trial Registry (www.sanctr.gov.za; DOH-27-042021-679). A reimbursement of ZAR 155 was issued to participants for their time. The labelling of records and all documents were designed to maintain patients’ confidentiality. All documents were cached in a facility and exposed to limited access and stored for at least two-years post-investigation. Clinical particulars were only shareable on receiving written consent form the participant, apart from information requisite for auditing by regulatory and oversight bodies.

## 3 Results

### 3.1 Participant characteristics

These results are presented in Table 1.

**Table 1:** Participant characteristics stratified by trial and HIV result.

	Trial 1			Trial 2 (Phase 1)			Overall		
	Negative	Positive	p	Negative	Positive	p	Negative	Positive	p
n	851	186		517	76		1368	262	
HIV Status = Positive (%)	0 (0.0)	186 (100.0)	<0.001	0 (0.0)	76 (100.0)	<0.001	0 (0.0)	262 (100.0)	<0.001
Gender = Male (%)	487 (57.2)	69 (37.1)	<0.001	333 (64.4)	37 (48.7)	0.012	820 (59.9)	107 (40.8)	<0.001
Age (median [IQR])	26.00 [22.00.34.00]	34.00 [30.00.39.00]	<0.001	26.00 [23.00.32.00]	32.00 [28.00.36.25]	<0.001	26.00 [22.00.33.00]	34.00 [29.00.39.00]	<0.001
Race (%)			0.854			0.496			0.575
Black	812 (95.4)	178 (95.7)		509 (98.5)	75 (98.7)		1321 (96.6)	254 (96.9)	
Coloured	30 (3.5)	6 (3.2)		3 (0.6)	1 (1.3)		33 (2.4)	7 (2.7)	
Indian	4 (0.5)	0 (0.0)		0 (0.0)	0 (0.0)		4 (0.3)	0 (0.0)	
Unknown	2 (0.2)	1 (0.5)		0 (0.0)	0 (0.0)		2 (0.1)	1 (0.4)	
White	3 (0.4)	0 (0.0)		5 (1.0)	0 (0.0)		8 (0.6)	0 (0.0)	
Education (%)			<0.001			0.206			<0.001
Diploma/Certificate	131 (15.4)	13 (7.0)		49 (9.5)	6 (7.9)		180 (13.2)	19 (7.3)	
High School	534 (62.7)	138 (74.2)		382 (73.9)	59 (77.6)		916 (67.0)	197 (75.2)	
None	4 (0.5)	2 (1.1)		12 (2.3)	0 (0.0)		16 (1.2)	2 (0.8)	
Primary School	22 (2.6)	13 (7.0)		23 (4.4)	7 (9.2)		45 (3.3)	20 (7.6)	
Tertiary	160 (18.8)	19 (10.2)		51 (9.9)	4 (5.3)		211 (15.4)	23 (8.8)	
Unknown	0 (0.0)	1 (0.5)		0 (0.0)	0 (0.0)		0 (0.0)	1 (0.4)	
Occupation (%)			0.642			0.594			0.970
Formal	77 (9.0)	12 (6.5)		25 (4.8)	6 (7.9)		102 (7.5)	18 (6.9)	
Informal	140 (16.5)	29 (15.6)		31 (6.0)	5 (6.6)		171 (12.5)	34 (13.0)	
Unemployed	631 (74.1)	144 (77.4)		459 (88.8)	65 (85.5)		1090 (79.7)	210 (80.2)	
Unknown	3 (0.4)	0 (0.0)		2 (0.4)	0 (0.0)		5 (0.4)	0 (0.0)	
Work Travel (%)			<0.001			0.125			<0.001
Month or longer	58 (6.8)	11 (5.9)		6 (1.2)	2 (2.6)		64 (4.7)	13 (5.0)	
No Travel	739 (86.8)	147 (79.0)		508 (98.3)	72 (94.7)		1247 (91.2)	220 (84.0)	
One Week	41 (4.8)	13 (7.0)		1 (0.2)	1 (1.3)		42 (3.1)	14 (5.3)	
Three Weeks	3 (0.4)	6 (3.2)		1 (0.2)	1 (1.3)		4 (0.3)	6 (2.3)	
Two Weeks	9 (1.1)	8 (4.3)		1 (0.2)	0 (0.0)		10 (0.7)	9 (3.4)	
Unknown	1 (0.1)	0 (0.0)		0 (0.0)	0 (0.0)		1 (0.1)	0 (0.0)	
HIV Test (%)			<0.001			<0.001			<0.001
0–3 Months	170 (20.0)	10 (5.4)		153 (29.6)	11 (14.5)		323 (23.6)	21 (8.0)	
3–12 Months	298 (35.0)	44 (23.7)		207 (40.0)	14 (18.4)		505 (36.9)	58 (22.1)	
More than 12 Months	269 (31.6)	108 (58.1)		120 (23.2)	39 (51.3)		389 (28.4)	148 (56.5)	
Never Tested	114 (13.4)	23 (12.4)		36 (7.0)	12 (15.8)		150 (11.0)	35 (13.4)	
Unknown	0 (0.0)	0 (0.0)		1 (0.2)	0 (0.0)		1 (0.1)	0 (0.0)	
Condom Use (%)			0.013			0.018			0.001
Never Had Sex	26 (3.1)	1 (0.5)		9 (1.7)	2 (2.6)		35 (2.6)	3 (1.1)	
No	120 (14.1)	33 (17.7)		0 (0.0)	0 (0.0)		120 (8.8)	34 (13.0)	
Sometimes	252 (29.6)	69 (37.1)		113 (21.9)	25 (32.9)		365 (26.7)	94 (35.9)	
Unknown	0 (0.0)	0 (0.0)		90 (17.4)	18 (23.7)		90 (6.6)	18 (6.9)	
Yes	453 (53.2)	82 (44.1)		305 (59.0)	31 (40.8)		758 (55.4)	113 (43.1)	
Anal Receptive (%)	51 (6.0)	15 (8.1)	0.367	178 (34.4)	33 (43.4)	0.161	229 (16.7)	48 (18.3)	0.593
Anal Insertive (%)	37 (4.3)	8 (4.3)	1.000	114 (22.1)	13 (17.1)	0.406	151 (11.0)	21 (8.0)	0.177
Vaginal Receptive (%)	234 (27.5)	67 (36.1)	0.023	137 (26.5)	22 (28.9)	0.756	371 (27.1)	89 (34.0)	0.029
Vaginal Insertive (%)	303 (35.6)	33 (17.7)	<0.001	223 (43.1)	18 (23.7)	0.002	526 (38.5)	52 (19.8)	<0.001
Oral Receptive (%)	130 (15.3)	16 (8.6)	0.026	164 (31.7)	22 (28.9)	0.723	294 (21.5)	39 (14.9)	0.019
Oral Insertive (%)	126 (14.8)	21 (11.3)	0.269	163 (31.5)	19 (25.0)	0.308	289 (21.1)	41 (15.6)	0.053
Sexual Partners (mean (SD))	1.50 (2.84)	1.46 (2.49)	0.887	1.35 (2.67)	1.05 (0.75)	0.328	1.44 (2.78)	1.43 (2.55)	0.950
Male Partners (mean (SD))	0.58 (1.75)	0.84 (1.23)	0.057	0.60 (2.52)	0.66 (0.83)	0.842	0.59 (2.08)	0.78 (1.13)	0.138
Female Partners (mean (SD))	0.92 (2.41)	0.63 (2.31)	0.134	0.75 (1.26)	0.39 (0.61)	0.015	0.86 (2.05)	0.65 (2.45)	0.148
Loss of Weight (%)			0.001			0.003			<0.001
No	412 (48.4)	69 (37.1)		470 (90.9)	60 (78.9)		882 (64.5)	130 (49.6)	
Unknown	325 (38.2)	72 (38.7)		0 (0.0)	0 (0.0)		325 (23.8)	72 (27.5)	
Yes	114 (13.4)	44 (23.7)		47 (9.1)	16 (21.1)		161 (11.8)	60 (22.9)	
Persistent Cold & Flu (%)	278 (32.7)	68 (36.6)	0.326	32 (6.2)	7 (9.2)	0.457	310 (22.7)	75 (28.6)	0.045
Diarrhoea and Fatigue (%)			0.134			0.273			0.018
No	700 (82.3)	143 (76.9)		511 (98.8)	74 (97.4)		1211 (88.5)	217 (82.8)	
Unknown	94 (11.0)	22 (11.8)		0 (0.0)	0 (0.0)		94 (6.9)	23 (8.8)	
Yes	57 (6.7)	20 (10.8)		6 (1.2)	2 (2.6)		63 (4.6)	22 (8.4)	
Night Sweats (%)			0.003			0.560			0.004
No	684 (80.4)	130 (70.0)		492 (95.2)	74 (97.4)		1176 (86.0)	205 (78.2)	
Unknown	65 (7.6)	16 (8.6)		0 (0.0)	0 (0.0)		65 (4.8)	16 (6.1)	
Yes	102 (12.0)	39 (21.0)		25 (4.8)	2 (2.6)		127 (9.3)	41 (15.6)	
Chills or Fever (%)			0.019			0.223			<0.001
No	378 (44.4)	68 (36.6)		512 (99.0)	74 (97.4)		890 (65.1)	142 (54.2)	
Unknown	448 (52.6)	105 (56.5)		0 (0.0)	0 (0.0)		448 (32.7)	106 (40.5)	
Yes	25 (2.9)	12 (6.5)		5 (1.0)	2 (2.6)		30 (2.2)	14 (5.3)	
Persistent SOB or Chest Pain (%)			0.110			1.000			0.006
No	342 (40.2)	61 (32.8)		508 (98.3)	75 (98.7)		850 (62.1)	136 (51.9)	
Unknown	448 (52.6)	105 (56.5)		0 (0.0)	0 (0.0)		448 (32.7)	106 (40.5)	
Yes	61 (7.2)	19 (10.2)		9 (1.7)	1 (1.3)		70 (5.1)	20 (7.6)	
Difficulty Eating (%)	36 (4.2)	9 (4.8)	0.853	21 (4.1)	4 (5.3)	0.547	57 (4.2)	13 (5.0)	0.678
White patches in mouth (%)	20 (2.4)	4 (2.2)	1.000	17 (3.3)	4 (5.3)	0.330	37 (2.7)	8 (3.1)	0.913
Bad breath (%)	59 (6.9)	24 (12.9)	0.010	33 (6.4)	8 (10.5)	0.221	92 (6.7)	32 (12.2)	0.003
Sore Throat (%)	71 (8.3)	13 (7.0)	0.656	22 (4.3)	5 (6.6)	0.373	93 (6.8)	18 (6.9)	1.000
Ulcers (%)	26 (3.1)	10 (5.4)	0.174	10 (1.9)	3 (3.9)	0.226	36 (2.6)	13 (5.0)	0.068
Dry Cough (%)	91 (10.7)	24 (12.9)	0.444	32 (6.2)	5 (6.6)	0.803	123 (9.0)	29 (11.1)	0.345
Wet Cough (%)			0.005			0.374			<0.001
No	363 (42.7)	62 (33.3)		509 (98.5)	74 (97.4)		872 (63.7)	136 (51.9)	
Unknown	448 (52.6)	105 (56.5)		0 (0.0)	0 (0.0)		448 (32.7)	106 (40.5)	
Yes	40 (4.7)	18 (9.7)		8 (1.5)	2 (2.6)		48 (3.5)	20 (7.6)	
Blood in Cough (%)			0.622			0.338			0.044
No	384 (45.1)	76 (40.9)		515 (99.6)	75 (98.7)		899 (65.7)	151 (57.6)	
Unknown	448 (52.6)	105 (56.5)		0 (0.0)	0 (0.0)		448 (32.7)	106 (40.5)	
Yes	19 (2.2)	4 (2.2)		2 (0.4)	1 (1.3)		21 (1.5)	5 (1.9)	
Heterosexual Partners (mean (SD))	1.24 (2.48)	1.14 (1.68)	0.592	1.15 (2.20)	0.87 (0.64)	0.267	1.21 (2.38)	1.15 (2.03)	0.705
Homosexual Partners (mean (SD))	0.25 (1.51)	0.32 (1.93)	0.586	0.20 (1.63)	0.18 (0.67)	0.921	0.23 (1.56)	0.28 (1.66)	0.653
STI History (mean (SD))	0.12 (0.35)	0.29 (0.47)	<0.001	0.10 (0.33)	0.20 (0.43)	0.027	0.11 (0.34)	0.27 (0.47)	<0.001
Assault History (mean (SD))	0.21 (0.43)	0.34 (0.47)	<0.001	0.14 (0.35)	0.20 (0.40)	0.162	0.18 (0.40)	0.30 (0.46)	<0.001

#### 3.1.1 Demographics

1036 participants were enrolled in trial one and 593 in trial two, 18% (186) and 13% (76) of whom were HIV positive respectively, yielding a prevalence of 16% across the total of 1630 patients enrolled. Overall, HIV positive patients had a higher median age (34 [IQR: 29–39] vs 26 [IQR 22–33], p < 0.001). Across the cohorts most patients were black (n = 1575 [97%]) with no statistically significant difference in HIV prevalence across racial groups. Although 927 (57%) participants were male, HIV was significantly less prevalent among males than females (107/927 [12%] vs 155/703 [22%], p < 0.001).

Most (1113/1630 [68%]) participants had a high school education, with HIV positive patients less likely to have had a post-high school education (391/1368 [29%] vs 42/262 [16%], p < 0.001) despite being of higher median ages. Most (1300/1630 [80%]) participants were unemployed with no statistically significant differences in HIV status across employment strata. Work-travel was significantly associated with HIV status with HIV positive participants more likely to travel for work (121/1368 [8.8%] vs 52/262 [16%], p < 0.001).

#### 3.1.2 Sexual behaviour

Condom use was significantly associated with HIV status with 55% (758/1368) of HIV negative participants and 43% (113/262) HIV positive participants reporting consistent condom use (p < 0.001). Reported rates of the different sexual intercourse behaviours were generally higher in the second trial than the first trial. Overall, vaginal receptive, vaginal insertive, and oral receptive intercourse were all significantly associated with HIV status. Specifically, vaginal receptive intercourse was reported more commonly in HIV positive participants as compared to HIV negative participants (89/262 [34%] vs 371/1368 [27%], p = 0.03) while vaginal insertive and oral receptive intercourse were reported less commonly in HIV positive individuals (52/262 [20%] vs 526/1368 [39%], p < 0.001 and 39/262 [15%] vs 294/1368 [22%], p = 0.02).

#### 3.1.3 Lifestyle

HIV positive participants had more commonly gone a year or more since their last HIV test as compared to HIV negative participants (183/262 [70%] vs 540/1368 [39%], p < 0.001). The mean number of total sexual partners were 1.44 (2.78) and 1.43 (2.55) among HIV negative and positive participants respectively with no significant differences between HIV status groups regardless of the sex or sexual orientation of the partner(s). HIV positive participants had higher mean numbers of both STIs and assaults within the last year (0.27 [0.47] vs 0.11 [0.34], p < 0.001 and 0.30 [0.46] vs 0.18 [0.40], p < 0.001 respectively).

#### 3.1.4 Symptoms

Tuberculosis symptoms including loss of weight, night sweats, a wet cough, chills or a fever, and blood in the cough were all significantly more common among HIV positive participants. Specifically, 60 (23%), 41 (16%) and 20 (7.6%) of 262 HIV positive participants reported loss of weight, night sweats, a wet cough, and chills or a fever as compared to 161 (12%), 127 (9.3%), 30 (2.2%) and 48 (3.5%) of the 1368 HIV negative participants respectively. Reporting no blood in the cough was more common among HIV negative participants (899/1368 [66%] vs 151/262 [58%], p = 0.03). Other symptoms associated with HIV positive participants included persistent shortness of breath (SOB) or chest pain, bad breath, diarrhoea, and fatigue and persistent cold or flu reported in 20 (7.6%), 32 (12%), 22 (8.4%), and 75 (29%) of the 262 HIV positive participants as compared to 70 (5.1%), 92 (6.7%), 63 (4.6%), and 310 (23%) of the 1368 HIV negative participants.

### 3.2 Model results

Initial AUROC results produced from 10-fold cross validation of the training split of the first trial are shown below in Table 2. Performance values are the average across all runs with 95% confidence intervals calculated from the result standard distribution. The training split was derived from a 70:30 random subset of the trial's responses. This was utilised to provide baseline estimates of each model group's performance, as well as to optimise any hyperparameters in relation to AUROC.

**Table 2:** 10-fold cross validation metrics from training split of trial one.

Model type	AUROC (95% CI)
Logistic Regression (glm)	71.56 (61.73–81.39)
Bayesian Logistic Regression (bayesglm)	76.40 (68.45–84.35)
Regularised Logistic Regression (glmnet)	79.24 (67.08–91.39)
Support Vector Machine with Linear Weights (svmLinearWeights)	78.88 (68.97–88.79)
Random Forest (ranger)	80.05 (67.63–92.47)
Gradient Boosted Tree Model (xgbTree)	82.67 (72.85–92.50)

**Fig. 2:**
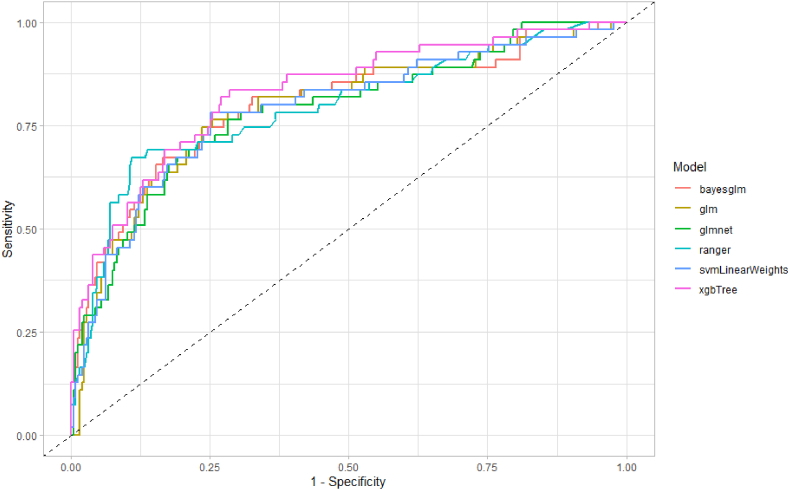
Receiver operator curves for all models evaluated using the holdout test portion of the initial data split from trial one.

Following this process, the optimised hyperparameters were used to fit each model on the full training split and subsequently evaluated on the holdout testing set. Performance metrics and associated receiver operator curves (ROCs) are shown in Fig. 2. AUROC values for the test set were substantially less disperse than those from model cross-validation, largely as a result of the logistic regressive models (glm and bayesglm) demonstrating a substantial improvement in performance. Irrespective of this alteration, the best performing architecture overall remains the gradient boosted tree model (xgbTree) with an AUROC of 82.84% (95% CI 76.48–89.21) (see Table 3).

**Table 3:** AUROC values for all models evaluated using the holdout test portion of the initial data split from trial one.

Model type	AUROC (95% CI)
Logistic Regression (glm)	79.15 (71.96–86.34)
Bayesian Logistic Regression (bayesglm)	79.91 (72.67–87.14)
Regularised Logistic Regression (glmnet)	78.47 (71.33–85.61)
Support Vector Machine with Linear Weights (svmLinearWeights)	78.92 (71.71–86.13)
Random Forest (ranger)	79.34 (71.99–86.69)
Gradient Boosted Tree Model (xgbTree)	82.84 (76.48–89.21)

**Fig. 3:**
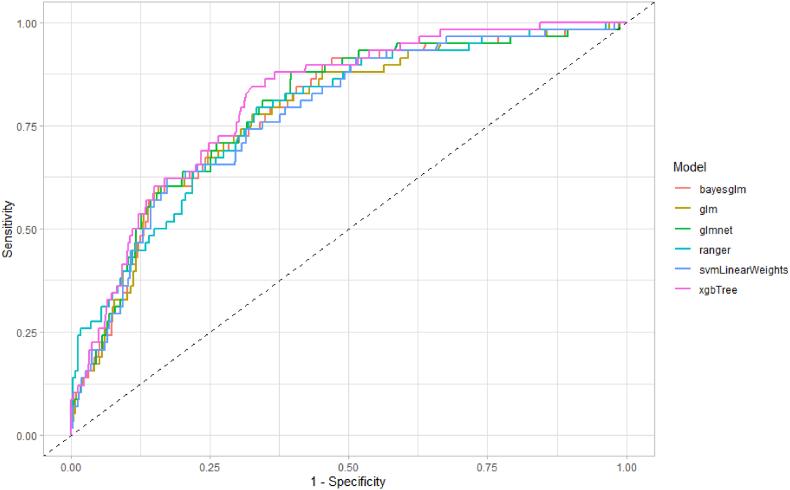
Receiver operator curves for all models trained using data from trial one and evaluated on the first phase of trial two.

Beyond simple holdout sample validation, the next evaluation sought to estimate model performance on an out-of-distribution, external validation scenario. This was achieved by evaluating the models trained from trial one's data on the entirety of phase one of trial two (see Table 4). While the question and response options matched across both trials, the geographical and demographic distributions differed substantially. Performance results and matched ROCs can be seen in Fig. 3. Despite these predictive challenges, model performance deteriorated only slightly with an average decline of only 1.16% (95% CI 0.13–2.20). Across all evaluations, gradient boosted decision trees averaged the greatest AUROC and as such were the best model for predicting HIV status.

**Table 4:** AUROC values for all models trained using data from trial one and evaluated on the first phase of trial two.

Model type	AUROC (95% CI)
Logistic Regression (glm)	77.78 (71.54–84.01)
Bayesian Logistic Regression (bayesglm)	78.14 (71.99–84.30)
Regularised Logistic Regression (glmnet)	78.89 (72.81–84.97)
Support Vector Machine with Linear Weights (svmLinearWeights)	77.58 (71.38–83.78)
Random Forest (ranger)	78.17 (71.93–84.42)
Gradient Boosted Tree Model (xgbTree)	81.09 (75.70–86.47)

### 3.3 Final model performance and interpretation

Final heterogenous model performance was evaluated using a mixed dataset consisting of trial one and the first collection window of trial two. Data was split into training, validation, and test subsets in a 70:20:10 ratio. The model was trained using the CatBoost python package. Both trials were shuffled and joined prior to splitting to enable an estimation of overall model performance on a heterogenous population. Performance evaluation employed a cut-off value of 0.12 on the holdout test-set as determined by a pre-determined sensitivity threshold of 90% on the validation-set. This model resulted in a sensitivity of 84% (95% CI 72–92), specificity of 71% (95% CI 67–76), and a negative predictive value (NPV) of 95% (95% CI 93%–96%), when evaluated on the hold-out test data (Table 5).

**Table 5:** Confusion matrix demonstrating boosted tree model prediction performance on holdout test-set with a binarization threshold of 0.12.

		Ground Truth	
		*Positive*	*Negative*
**Prediction**	*Positive*	46	74
	*Negative*	9	181
		***Precision/PPV:*** 0.38	***NPV:*** 0.95
	***Accuracy:*** 0.73	***Sensitivity:*** 0.84	***Specificity:*** 0.71

#### 3.3.1 Number needed to treat

The final mode was tested on an unseen dataset in the form of the three month follow up data from trial two (phase two). The predictions are tabulated against the ground truth as a two-by-two table (Table 6). This is used to evaluate the predictive performance of the model for seroconversion at three months. All individuals that acquired HIV were flagged by the model at the first visit with a risk

**Table 6:** Two-by-two table demonstrating boosted tree model prediction performance on patients returning for repeat testing in trial two.

		Ground Truth	
		*Positive*	*Negative*
**Prediction**	*Positive*	4	38
	*Negative*	0	185

value greater than the threshold. If these 42 individuals marked as being at risk were placed on pre-exposure prophylaxis this would mean the treatment of 10 (95% CI 5–155) individuals would have been required to prevent one seroconversion within three months.

#### 3.3.2 Model interpretation and variable importance

**Fig. 4:**
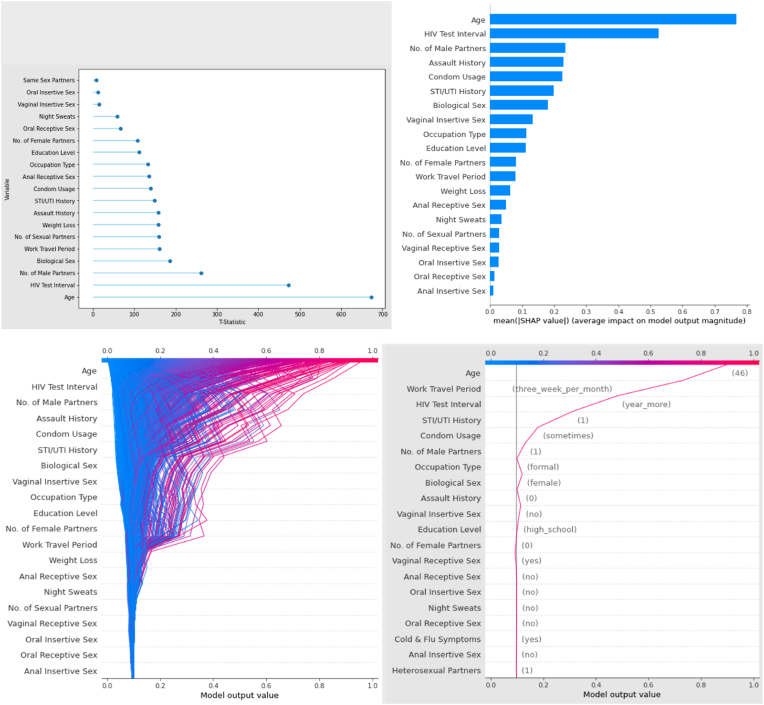
(A): T-Statistic measuring model performance improvement when including the variable in question compared to random shuffling of the column for the boosted tree model (PIMP methodology). Fig. 4 (B): Mean SHapley Additive exPlanations (SHAP) value measuring the average impact on model output of each variable. Fig. 4 (C): Example of using the SHAP methodology to demonstrate the contribution of each predictive feature to the overall model risk output value. The degree of inflection for each variable indicates the strength to which it influences the final result. Each line plot represents an individual from the dataset. Fig. 4 (D): Further example of using the SHAP methodology to demonstrate the contribution of each predictive feature to the overall model risk output value. The line plot represents the decision contribution of each variable for a single example individual. Figures are index left-to-right, top-to-bottom.

The most important variables in determining the risk for an individual, as ascertained using the PIMP methodology, are demonstrated in Fig. 4A and Table 7. Using this approach, the most significant variable by a wide margin is the age of the individual (t = 673.0), with the second most substantial being the length of time since the last HIV test (t = 472.1). Following on from this, the number of male sexual partners (t = 261.6), as well as the biological sex of the respondent (t = 186.7) have the next most substantial impact on risk. Sexual behaviour characteristics such as having previously had a urinary tract or sexually transmitted infection (UTI/STI) (t = 148.5), pattern of condom usage (t = 140.1), and type(s) of sexual intercourse also play a role. Other included factors are socioeconomic predictors, such as the level of education (t = 111.4) or occupation (t = 132.5) of an individual, and symptomatology such as the presence of night sweats (t = 59.2) or recent loss of weight (t = 158.8).

**Table 7:** Final model feature importance values derived from the PIMP method using 1000 shuffle replications per distribution. Distance is measured via the t-statistic and p values are derived from the overlap between distributions.

Feature	T-Statistic	p value
**Age**	673.0465	<0.001
**HIV Test Interval**	472.1002	<0.001
**No. of Male Partners**	261.5906	0.0004
**Biological Sex**	186.6529	0.0091
**Work Travel Period**	160.7948	0.0232
**No. of Sexual Partners**	159.4615	0.2994
**Weight Loss**	158.7563	0.1576
**Assault History**	158.4471	0.0225
**STI/UTI History**	148.4863	0.0304
**Condom Usage**	140.0723	0.0389
**Anal Receptive Sex**	135.3916	0.2310
**Occupation Type**	132.517	0.1605
**Education Level**	111.3594	0.1292
**No. of Female Partners**	107.565	0.2096
**Oral Receptive Sex**	66.62	0.4606
**Night Sweats**	59.2416	0.3769
**Vaginal Insertive Sex**	14.2625	0.4372
**Oral Insertive Sex**	12.427	0.4872
**Same Sex Partners**	7.6071	0.4969

Fig. 4D presents a summary SHAP as an additional estimate for aggregate explanation of variable importance. Age, duration since last HIV test, number of male sexual partners, a history of assault, and reporting weight loss were identified as the top five most impactful variables on model output.

Fig. 4C and D presents further plots of variable importance ascertained using the SHAP methodology. Fig. 4C presents the individual contributions of each observation in the hold-out test dataset to the aggregate SHAP estimation (Fig. 4B) while Fig. 4D presents an example of a single prediction decomposed. In this example, the final model estimation of a high-risk score close to 1 is substantially driven by the individual's age, work travel pattern, long HIV testing interval, STI history, and occasional condom usage.

## 4 Discussion

Having the ability to handle great quantities of population and health-related data swiftly, machine learning has the potential to identify patients who are at high risk of contracting HIV. This study was undertaken to assess the viability of ML in the identification of such patients particularly in LMICs, using patient data collected from digital surveys administered directly to potential patients as opposed to secondary analysis of a pre-existing electronic database. Participants who were not known to be HIV positive were enlisted in two consecutive trial studies.

### 4.1 Study characteristics

Trial one and trial two had similar distributions of baseline demographic characteristics. Overall, there are notably high levels of unemployment, a feature possibly attributable to the timing and incentive structure of the studies. There were more differences between the studies with respect to sexual behaviour with noticeably higher rates of all forms of intercourse in trial two which was likely a product of a flaw in the digital survey used in trial two that allowed participants to “select all”. If there were non-random use of the select all function this could have biased the study results. Lifestyle characteristics were similar across both studies. An important difference between symptoms reported between the studies was that in the second trial respondents had to answer all the questions and thus there was no “unknown” field across trial two. This again could represent a source of bias if there was differential non-response. The HIV prevalence across trial one and two was relatively similar (18% and 13% respectively). This is in keeping with the estimated 13.4% HIV prevalence across South Africa in 2019 [29]. One would have expected the prevalence in the studies to be lower given being known with HIV infection was one of the exclusion criteria, but this may have been countered by the intentional selection of high prevalence settings with HIV prevalence statistics that vary from 13.1% (Hammanskraal, Tshwane) to 27.8% (Isipingo, Ugu) [30].

### 4.2 Associations with HIV status

#### 4.2.1 Demographic characteristics

Key baseline demographic associations with HIV included female sex, older age, lower levels of education, and longer durations of work travel. These findings are in keeping with findings from other studies in South Africa [[31], [32], [33]]. The importance of demographic characteristics in HIV risk quantification is reflected in both the final ML model variable importance estimators with age being the most important variable using both the PIMP (t = 673.0) and global SHAP methodology. The importance of these characteristics is well exemplified in the single prediction decomposition in Fig. 4d in which work travel and age were the greatest contributor to the example individual's risk.

#### 4.2.2 Sexual behaviour

Condom use is a well described and quantified behavioural intervention to mitigate HIV infection risk with data to suggest around an 80% efficiency at preventing transmission [34]. This is in keeping with the significantly lower reported rates of consistent condom use among HIV positive participants across both trials. The importance of this behavioural intervention is again reflected in both the PIMP and global SHAP variable importance assessments as well as highlighted in the example single prediction decomposition SHAP in which “sometimes” condom use is the 4th most important contributor to the example individual's risk. In addition, receptive vaginal sex was noted to be the most common form of sexual intercourse among HIV positive participants with a significant difference between the groups. This is in keeping with HIV epidemic within South Africa where heterosexual females are noted to be at highest risk of HIV infection for various social and biological reasons [35,36]. However, within the modelling, this appears to be captured by the number of male partners (t = 261.6). This is also reflected in Fig. 4d where one male sexual partner was the 6th most important contributor to the example individual's risk.

#### 4.2.3 Lifestyle

A longer duration since the participant's last HIV test as well as higher numbers of STIs and experiences of assault within the last year were the most important lifestyle related variables. The significance of the duration since last HIV test highlights the importance of broad access to HIV counselling and testing services and the need to expand these services to missed groups [37]. The association with recent history of STIs is in keeping with the literature and has a known biological basis [38]. It is useful to note that HIV and STIs are likely co-linear which would be a challenge for traditional modelling strategies but allows for synergy in prediction of each in this case. The final model places high importance on both STI history and, particularly, duration since last HIV test as highlighted in the PIMP and global SHAP and once again is reflected in the relative importance of the presence of a longer duration since last HIV test and a positive STI history in the high-risk assessment of the example individual reflected in Fig. 4b.

#### 4.2.4 Symptoms

The higher prevalence of tuberculosis symptoms among HIV positive participants is unsurprising given the well documented higher rates of the disease among HIV positive patients regardless of HIV progression [39]. The other symptoms reported more commonly are typically associated with less advanced stages of HIV infection [40] and highlights the potential value of this tool in identifying patients with HIV at earlier clinical stages before they present to a healthcare facility with an AIDS-defining condition. Generally, symptomatology had less influence on the model's predictions although it is noted that a few tuberculosis symptoms featured more prominently in the SHAP variable importance assessment.

### 4.3 Model comparison and final model performance

Model comparison revealed that, despite their simplicity, a large proportion of predictive performance was captured by logistic classification models. These models enable greater explanatory ability at the expense of some performance due to the lack of automated variable interaction among other techniques. However, the final model selected was a gradient boosted decision tree as it provided a substantial increase in performance. The superior classification capacity of ML over traditional modelling previously noted by other authors [14] was similarly noted in this study. In addition, if data collection were to be expanded to include additional geospatial or temporal factors, this class of predictive models would likely to scale rapidly in performance as was found by Orel et al. where an XGBoost model performed best for their data which included longitude, latitude, and altitude [15].

The AUROC measures of the models we evaluated were similar to those of other authors [14,19,20]. Our model and chosen cut-off performed better prospectively, flagging 100% (4/4) of incident HIV cases as high risk as compared to 38.6% (32/83) [19] however, on the hold-out data from the final model partition our model performed almost identically, flagging 38.3% (46/120) of positive participants as high risk. These findings highlights that the use of digital survey data can produce ML models that are comparable to those built using larger datasets.

### 4.4 Clinical implications

#### 4.4.1 Modifying the public health response

This study identifies two important features of the use of ML models in HIV risk assessment. First, the global (aggregate) variable importance assessments (Fig. 3, Fig. 4) highlight the important components of population-level risk. Given enough data one would be able to model HIV risk in different communities and use the variable importance estimates to identify the largest local contributors to risk and adjust the public health response accordingly. For example, should limited condom use and many STIs be contributing significantly to a community's risk, specific action could be taken to address those two issues. Similarly, the forced SHAP plot (Fig. 4d) helps to quantify the specific components of an individual's risk. If this was thought of in terms of modifiable risk (e.g., condom use) and non-modifiable risk (e.g., age) this could help guide personalised counselling services towards either behaviour change strategies or PrEP.

#### 4.4.2 PrEP decision support

Much of the ML work done in HIV thus far has been focused on supporting and guiding PrEP treatment decisions [[19], [20], [21]]. In particular, Zheng et al. used machine learning to maximize the number of seroconversions prevented while minimizing the number of people on PrEP and showed that one can use such an approach to offer individualized PrEP decision support in resource limited settings [21]. This is in keeping with data from another study that showed that ML can identify an “at risk” population that is small enough to offer directed preventative services [15]. Given the relative ease of use of the digital survey used in this trial, such a survey and associated risk assessment algorithm could be used to identify the at-risk individuals who would benefit most from being offered PrEP while tailoring the risk-threshold to the resources available. The estimated number needed to treat of 10 individuals to prevent one infection at three months emphasizes how successful such an approach could be.

### 4.5 Future directions

To our knowledge this is the first study of its kind to develop a digital survey of socio-behavioural questions and use it as a primary data collection tool to build a ML model to estimate HIV risk. It is anticipated that such a tool could potentially be integrated into the clinical workflow to enhance the public health response to HIV at both the population and individual level as well as to decision support around PrEP initiation. Such an approach would provide a continuous source of data, which if linked to ground truth could be used to validate and further enhance the predictive capacity of the model. In addition, given the co-linearity, such a platform could be used to assist with screening for tuberculosis and STI symptoms in the general population. Finally, the inclusion of geospatial, temporal, or local incidence data may further enhance the predictive ability of the model.

### 4.6 Limitations

The major limitation of this study concerns the combination of data from two separate studies. While this process was made simpler by the fact that the second trial used a refined version of the survey from the first trial and every effort was made to ensure consistency between the datasets, such an approach still carries the risk of miscoding a response. Similarly, the “select all” option for different intercourse options in trial two as well as the ability not to select an option to some questions in trial one could have introduced bias if there was differential selection of either of these options by HIV status. Additionally, it is to be noted that all variable importance methodologies have recognised limitations and neither of the two presented here can be certain to fully capture the portion of risk attributable to a particular variable. Finally, the non-random sampling strategies employed are also a limitation to the study as they may also have introduced bias.

## 5 Conclusion

This study has highlighted the synergies present between mHealth methodologies and ML in the field of HIV risk prediction. It has been demonstrated that a viable ML model can be built using digital survey data with interlinking potential utility in directing health resources, including PrEP, towards the areas of greatest potential benefit. It has been shown that such an approach could be viable in LMICs, such as South Africa, in which they are most needed.

## Declaration of competing interest

The authors declare the following financial interests/personal relationships which may be considered as potential competing interests: Samanta Lalla-Edward reports financial support was provided by 10.13039/100000865Bill & Melinda Gates Foundation.
